# Scoping the Priorities and Concerns of Parents: Infodemiology Study of Posts on Mumsnet and Reddit

**DOI:** 10.2196/47849

**Published:** 2023-11-28

**Authors:** Christopher Thornton, Kate Lanyi, Georgina Wilkins, Rhiannon Potter, Emily Hunter, Niina Kolehmainen, Fiona Pearson

**Affiliations:** 1 National Institute for Health and Care Research Innovation Observatory Population Health Sciences Institute Newcastle University Newcastle Upon Tyne United Kingdom

**Keywords:** childhood, child, toddler, infant, behavior, parent, parenting, topic modeling, data mining, social media, infodemiology, Reddit, web-based forum, well-being, children, data, family health

## Abstract

**Background:**

Health technology innovation is increasingly supported by a bottom-up approach to priority setting, aiming to better reflect the concerns of its intended beneficiaries. Web-based forums provide parents with an outlet to share concerns, advice, and information related to parenting and the health and well-being of their children. They provide a rich source of data on parenting concerns and priorities that could inform future child health research and innovation.

**Objective:**

The aim of the study is to identify common concerns expressed on 2 major web-based forums and cluster these to identify potential family health concern topics as indicative priority areas for future research and innovation.

**Methods:**

We text-mined the r/Parenting subreddit (69,846 posts) and the parenting section of Mumsnet (99,848 posts) to create a large corpus of posts. A generative statistical model (latent Dirichlet allocation) was used to identify the most discussed topics in the corpus, and content analysis was applied to identify the parenting concerns found in a subset of posts.

**Results:**

A model with 25 topics produced the highest coherence and a wide range of meaningful parenting concern topics. The most frequently expressed parenting concerns are related to their child’s sleep, self-care, eating (and food), behavior, childcare context, and the parental context including parental conflict. Topics directly associated with infants, such as potty training and bottle feeding, were more common on Mumsnet, while parental context and screen time were more common on r/Parenting.

**Conclusions:**

Latent Dirichlet allocation topic modeling can be applied to gain a rapid, yet meaningful overview of parent concerns expressed on a large and diverse set of social media posts and used to complement traditional insight gathering methods. Parents framed their concerns in terms of children’s everyday health concerns, generating topics that overlap significantly with established family health concern topics. We provide evidence of the range of family health concerns found at these sources and hope this can be used to generate material for use alongside traditional insight gathering methods.

## Introduction

Innovation for health and care is increasingly supported by bottom-up approaches to priority setting [1-4], reflecting the importance of allowing the views and concerns of the intended beneficiaries to influence the aims of innovation [5]. Traditional priority-setting methods emphasize collaboration with the intended beneficiaries and can be a lengthy process, involving 15 steps in some cases [6]. However, often a more rapid approach to priority setting may be desired, and so, here we present methods to rapidly identify the priorities and family health concerns of parents using web-based forum data.

The views of parents and children have made important contributions to setting priorities and the conduct of childhood research [7-10]. Parents are in most cases the primary caregivers for their children and are well placed to provide signals about the needs and concerns related to their children’s health and well-being. This enables the identification of gaps between current child health priorities and the main concerns of parents, which can help to inform the future direction of research and innovation [11].

Social media sites and web-based forums contain the thoughts, questions, opinions, and concerns of parents on a range of topics, where they initiate and drive discussions. Anonymous social media content can augment traditional methods [7,10] used to assess the views of parents and caregivers on a wide range of topics. Novel analysis methods have previously been used to aggregate social media–sourced stories relating to parental attitudes toward vaccine use in children [12], to capture the experiences of new fathers [13], to understand caregiver burdens [14], and to understand the concerns of foster families during the COVID-19 pandemic [15].

Similar methods can be used to identify the unmet needs of families more generally. In this way, it will become feasible to rapidly scope the thoughts and concerns of parents regarding parenting and the health and well-being of their families. Identifying parents’ views in this manner draws from a variety of first-hand experiences and expressions of specific needs, providing a rich source of data on family health concerns for which sufficient information or solutions are likely yet to be found. Here, we aim to provide valuable insight that can be used to identify unmet needs and inform innovation priorities [7-10].

## Methods

### Ethical Considerations

This study was approved (REF 2417/25036) by the Faculty of Medical Sciences Research Ethics Committee, part of Newcastle University's Research Ethics Committee.

### Data Sources

Mumsnet is a UK-based forum that is widely used to seek advice or emotional support [16] through anonymous posts, usually in the form of a question, and responses from other users. The website is not restricted to discussion of parenting, but there is a specific forum for parenting questions. Data collected from Mumsnet have previously been used to describe the views of parents (particularly mothers) and answer a wide range of research questions [17-20].

Reddit is a large and widely used general-purpose social media platform. It contains subreddits (subforums) for a wide range of topics, including several on parenting, with r/Parenting the largest of these parenting subreddits. Reddit has increasingly been recognized as an important resource for researchers [21,22], including research into childhood and parenting [14,23].

### Data Collection and Cleaning

Posts were collected from the r/Parenting subreddit between January 1, 2010, and February 1, 2022, using a custom Python (version 3.8.5; Python Software Foundation) script which called the Pushshift application programming interface [24] and from the parenting forum on Mumsnet between April 19, 2001, and March 5, 2022, using a custom Python web scraping script. This captured all posts made on these forums between the dates specified. Posts from both sources were then cleaned by removing placeholders for deleted posts (eg, “[removed]” or “Message withdrawn at poster's request.”), and a spam post containing a single word was posted at a high frequency on one of the forums. We also standardized the spelling and language to British English (eg, diaper to nappy) and standardized apostrophe-like syntax to a single character. Cleaning was performed using custom Python code available at [25].

### Data Preprocessing

We then preprocessed the data, again using custom Python code, to create a corpus for the topic model. This involved removing stopwords (we used the English language stopwords from the Natural Language Tool Kit Python library [26]). We also applied the standard preprocessing provided by the gensim Python library [27] that removes most punctuation and numeric characters, removes words with fewer than 4 characters, converts all to lowercase, and stems all words. Bigrams (common 2-word phrases) were also detected using the gensim Phrases class and added to the corpus as single entities. The final corpus was composed of the preprocessed posts after words with fewer than 50 occurrences or words that occur in more than 50% of all posts removed.

### Modeling Indicative Topics of Common Concern

Frequently discussed family health concern topics were detected using latent Dirichlet allocation (LDA), a generative statistical model, previously described in detail elsewhere [28]. This involved first compiling a dictionary of all words used in the corpus of texts (social media posts in this case), with each word represented by a number. Each post was then encoded as a set (unordered list) of the words it contains. The LDA process calculates the probability of a given model generating the set of posts we have. This probability is then used to evaluate the model against the data, facilitating the estimation of an optimal model. In the case of gensim, an online variational inference algorithm described here [29] is used to perform the optimization. To perform LDA topic modeling, we passed our corpus of texts (stored as vectors of word IDs) to the constructor of LdaMulticore class provided by the gensim library. Hyperparameters were set as follows: passes=20, chunksize=1000, decay=0.5, offset=64, iterations=1000, and gamma threshold=0.001. α was set to a fixed symmetric prior to 1/number of topics. To estimate the optimal number of topics, we performed a scan of the topic number parameter (with a range of 2 to 34 topics), using coherence as our evaluation metric. We repeated this for 10 random initial starting states and used the number with the highest mean coherence. The coherence was assessed by calculating a composite measure known as C_v_, previously shown to correlate well with human-assessed topic coherence [30].

Using the topic distribution calculated for each post, we estimated the probability of each post belonging to a topic. Posts were considered as belonging to a topic if the probability was greater than 75%.

### Labeling and Validation of Topics of Common Concerns

Topics were interpreted in 2 ways. First, the top 4 words associated with the topic were identified and used to name the topic. This association was calculated using the equation *a*=log (∅)+log (∅)/*P*, where ∅ is the probability of the word given the topic, *P* is the probability of the word appearing in the corpus, and *a* is the association.

Second, to further facilitate the interpretation of topics as indicative parenting or family health priority areas, we applied a 2-step qualitative analysis to a subset of 50 posts belonging to each topic. The first step was to identify parenting concerns without reference to any prior categories, and the second step was to place each post, if possible, into one or more of the parenting question categories identified by Lavigne et al [31]. The posts analyzed were randomly selected from all posts with a greater than 75% probability of being generated by the topic. The full analysis procedure is described below:

- Posts were distributed among 4 researchers.
- Researchers read through each post.
  They identified and noted down each parenting or family health concern (if any) the post contains.
  They also identified whether the post could be categorized into one or more of the 11 question categories identified by Lavigne et al [31].
  Posts were collated back together, discussed by the researchers, and then collective decisions were made on any that were unclear.
- The parenting or family health concerns occurring 3 or more times within the sample of 50 posts were taken forward as a signal of specific areas of concern. This threshold was selected because it gave a greater than 90% (93.2%) probability of the concern occurring in at least 1% of all posts in the topic (using a proportions z test to determine the likelihood that the proportion 3/50 is greater than 0.01; *P*=.07).
- The coherency of each topic was assessed manually by an analysis of the parenting concerns identified. If there was a single concern, or the concerns were closely related, the topic was deemed coherent. If there were multiple concerns that were distinct but with clear connections, the topic was deemed to have related clusters. If there were multiple concerns with no obvious connections, then the topic was deemed to have unrelated clusters.

## Results

### Topic Model

**Figure 1:**
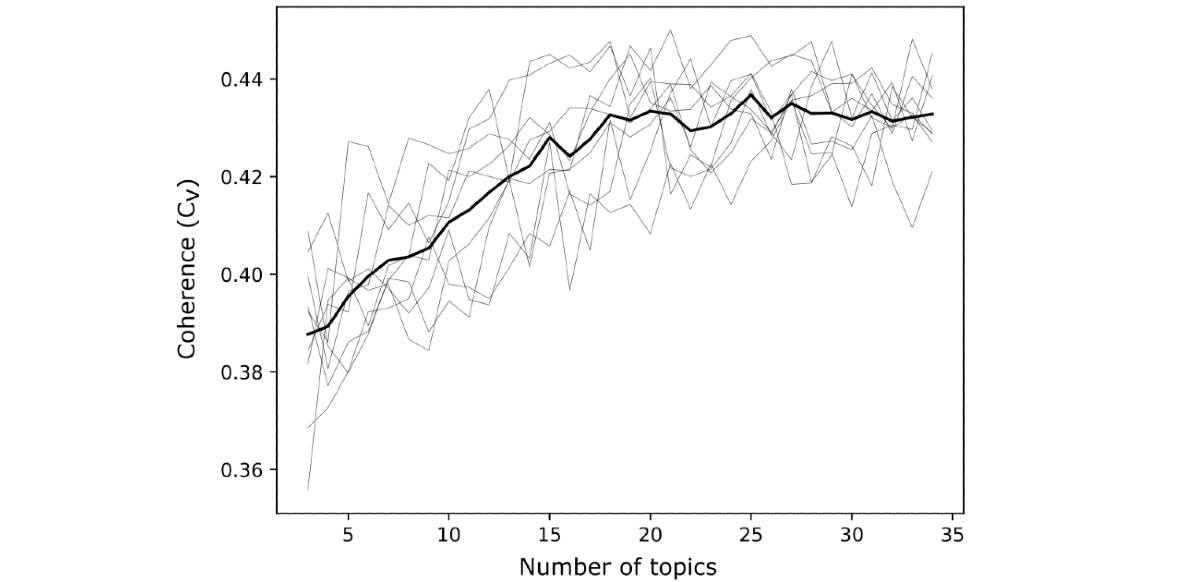
The coherence of the topic model as the number of topics increases. The figure shows the 10 repeats performed for each number of topics (thin line) and the mean of all repeats (thick line).

We collated 169,694 posts in total, 99,848 from Mumsnet and 69,846 from the r/Parenting subreddit. Of these, 153,182 remained after cleaning and were provided to the LDA topic model for estimation. Figure 1 shows the overall coherence of the topic model with an increasing number of topics. We found that the model coherence improved between 4 and 25 topics, after which it plateaued. A 25 topic model had the highest average coherence across repeats and so was selected as the model to take forward for further analysis.

Table 1 describes the 25 topics, showing the number of posts assigned to a topic, the 4 words with the highest association with the topic, the specific areas of concern within the topic identified through the inductive content analysis, and the manual assessment of whether the sample of posts from each topic was coherent.

**Table 1:** The 25 topics identified by the optimal LDA^a^ model alongside the inductively identified parenting or family health concerns.

Posts, n (%)	LDA topic	Inductively identified areas of specific parenting or family health concerns	Coherence of concerns
4295 (12.18)	sleep+night+wake+feed	Bottle, breastfeeding, crying, nap, regression, routine, sleeping in parents’ bed, sleeping routine, waking up at night	Related clusters
2482 (7.04)	size+wrap+buggi+cloth	Affordability, cloth nappies, clothing for carrying baby, clothing temperature	Related clusters
2435 (6.9)	potti+train+potti_train+toilet	Potty/toilet training	Coherent
2245 (6.36)	food+milk+meal+drink	Food ideas, formula, picky eater, puree food, water, weaning, whole food	Coherent
2132 (6.04)	pregnant+pregnanc+birth+famili	Family issues, having another child, leaving child	Related clusters
2053 (5.82)	work+nurseri+childcar+studi	Childcare, financial advice, going back to work, studying	Related clusters
1669 (4.73)	nappi+teeth+dispos+leak	Cloth nappies, leaking nappies, washing nappies	Coherent
1632 (4.63)	parent+wife+father+relationship	Depression, mental health, co-parenting, divorce, grandparents, parenting, parenting style, authoritarian parent	Related clusters
1521 (4.31)	doctor+sick+cough+hospit	Child feeling ill, Covid concerns, fever	Coherent
1521 (4.31)	plai+friend+school+swim	Activities, fears, friends	Related clusters
1420 (4.03)	parti+birthdai+seat+christma	Advice on gifts, affordability, birthdays, Christmas, gifts for others’ children, presents, car seat safety, car seats, growing children, travel	Unrelated clusters
1420 (4.03)	feel+like+guilti+feel_guilti	Bad parent worries, feeling guilty, finding it difficult being a parent, feeling overwhelmed, frustration, hoping it will get better	Coherent
1409 (3.99)	told+behaviour+tell+punish	Bad behavior, behavioral problems, domestic abuse, sibling violence, intimate partner violent interactions, separated father	Related clusters
1291 (3.66)	school+book+teacher+read	Adult course, book recommendations, school admissions, tutoring	Coherent
1281 (3.63)	reflux+poop+constip+formula	Allergy, breastfeeding, colic, constipation, digestive issues, milk, milk allergy, reflux, stomach problems	Coherent
958 (2.72)	word+speech+autism+delai	Autism, developmental concerns, education questions, speech, speech issues	Coherent
923 (2.62)	english+speak+languag+French	Activities, development, friends, language	Coherent
887 (2.51)	scream+bedtim+tantrum+routin	Bedtime, bedtime routine, crying/screaming, daily routines, dummy removal, naps, screaming/crying	Coherent
797 (2.26)	girl+phone+friend+messag	Child internet concerns, monitoring, social media, social media harm, stranger interactions, trust	Related clusters
716 (2.03)	room+bedroom+chair+mattress	Affordability, baby sleeping space, bedroom, sleeping arrangements, bouncy chairs, highchair, living arrangements, safety, toy storage	Coherent
534 (1.51)	game+watch+video+movi	Films, hobbies, sport, video games, YouTube	Coherent
518 (1.47)	hair+wash+clean+bath	Bath, cleaning, hair, hygiene, nails, potty training, recommendations	Coherent
511 (1.45)	daycar+monei+covid+mask	Activities, childcare, Covid, money, responsibility	Related clusters
313 (0.89)	head+roll+crawl+stair	Crawling, development, movement, rolling, standing, walking, equipment, falling, illness, injury fears, stairs	Coherent
313 (0.89)	door+open+mommi+lock	Child scared, children being funny, explaining death to child, explaining sex to child, pet issues	Related clusters

^a^LDA: latent Dirichlet allocation.

**Figure 2:**
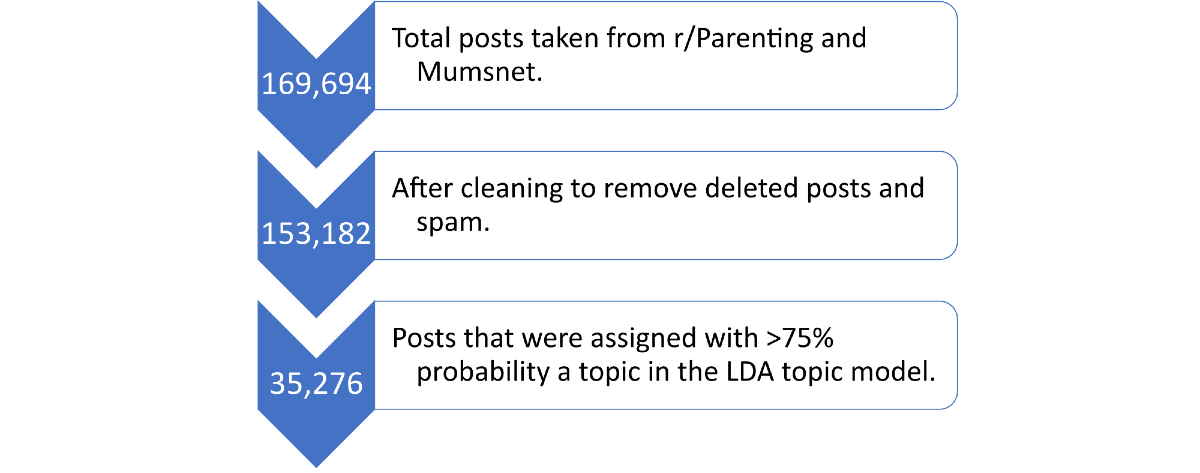
The number of posts from initial collection to final analysis. LDA: latent Dirichlet allocation.

**Figure 3:**
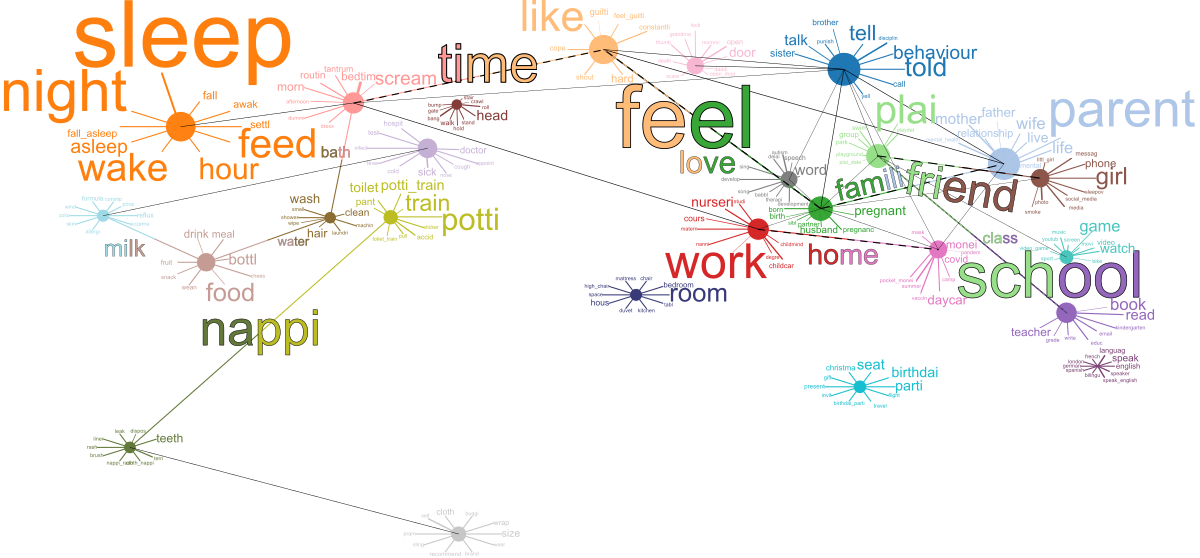
The topics identified by the latent Dirichlet allocation process—each circle represents a topic, linked to the 10 most common words used in the topic. The size of the circle represents the size of the topic, and the size of the word represents the frequency of its use. Topics are linked together if their word frequency distribution has a cosine similarity above 0.4 (black line) or if they share a top 10 word (colored line) or both (dashed line).

**Figure 4:**
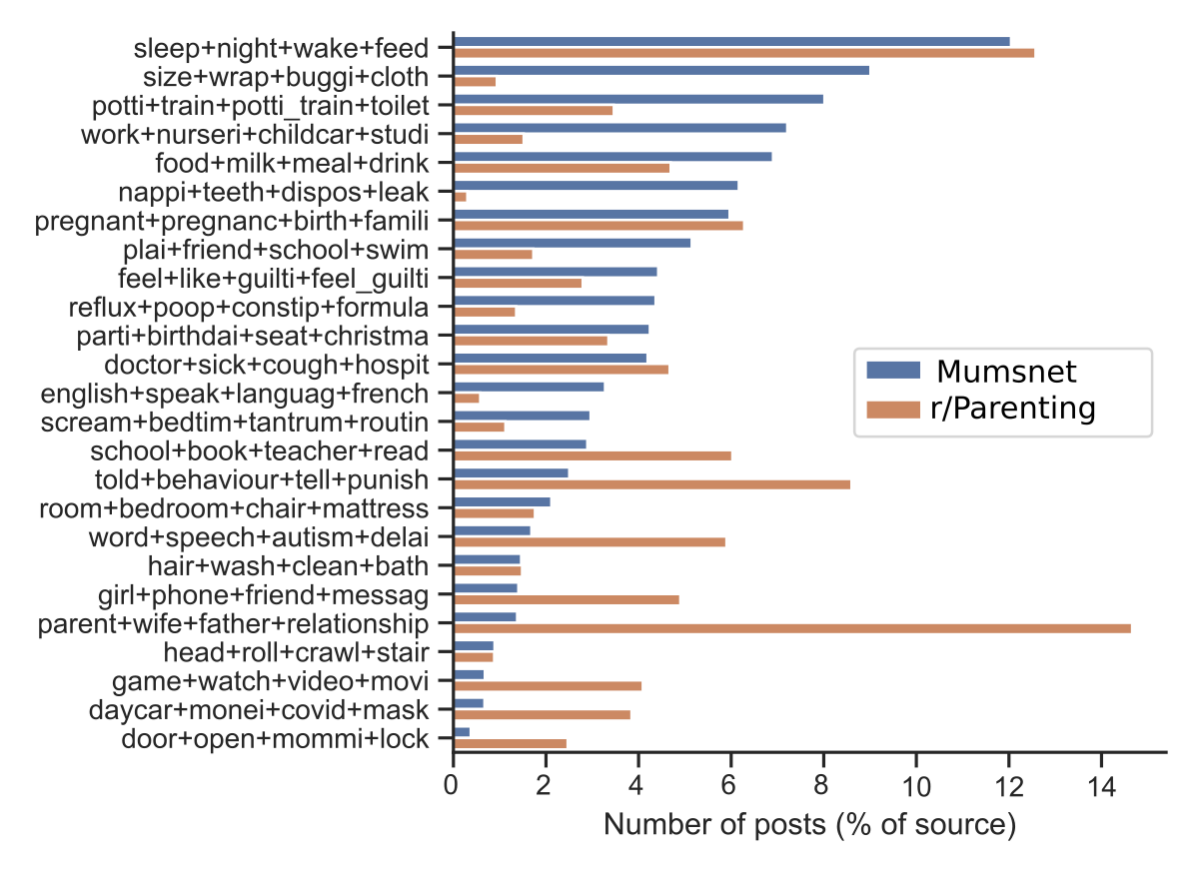
The frequency of topic occurrence within each source media. The number of posts is normalized by the total contributed by that source.

We found that we could assign 35,276 (23%) posts to a topic with greater than 75% probability and that when we used this to filter posts, topics contained between 313 posts (head+roll+crawl+stair and door+open+mommi+lock) and 4295 posts (sleep+night+wake+feed). Figure 2 shows the number of posts included at each stage of the processing pipeline. Figure 3 shows each topic found by the model, positioned according to a 2-dimensional projection of the distance between topics as calculated by the LDAvis library [32]. The 10 most common words in each topic are shown along with words shared between topics. In the final collection, Mumsnet contributed 26,648 posts, while r/Parenting contributed 8628 posts. Figure 4 shows the proportion of posts from each platform that have been assigned to each topic. This allows us to see that some topics are more commonly discussed on 1 platform, for example, potty training and nappy changing topics (potti+train+potti_train+toilet, size+wrap+buggi+cloth, and nappi+teeth+dispos+leak) are more commonly discussed on Mumsnet, while topics on domestic abuse, divorce, and parenting style (told+behaviour+tell+punish and parent+wife+father+relationship) are more common on Reddit. Topics such as sleep (sleep+night+wake+feed), pregnancy (pregnant+pregnanc+birth+famili), or visiting the doctor (doctor+sick+cough+hospit) are equally common on both platforms.

### Interpretation of Topics

**Figure 5:**
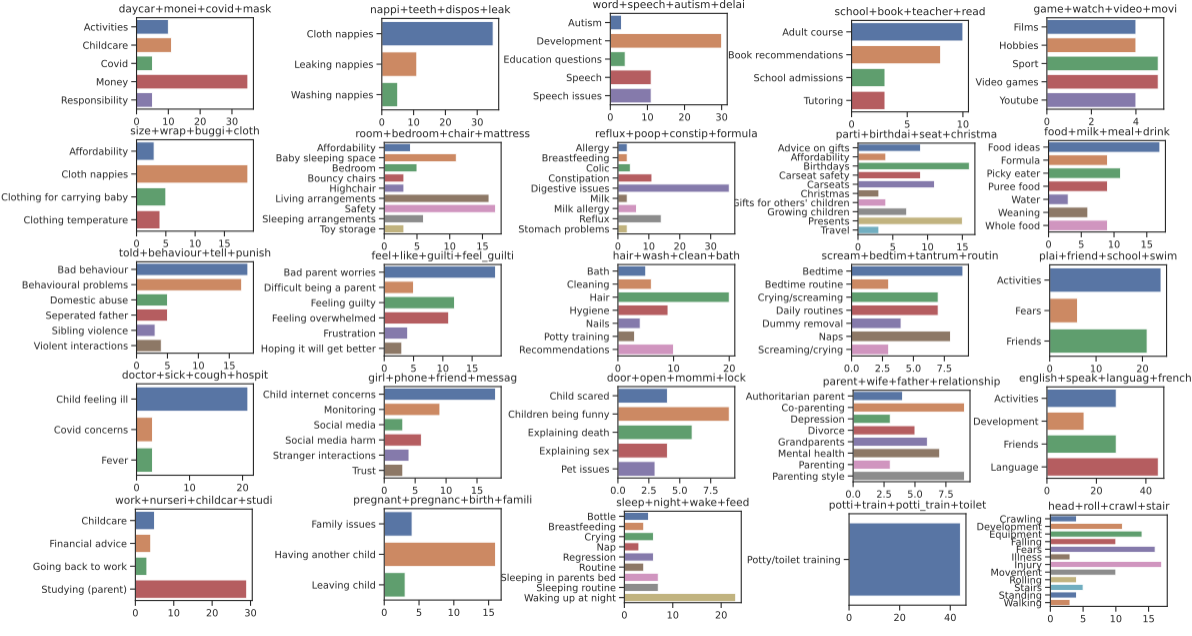
The categories identified manually from within posts sampled from each topic.

From the topic validation, we found that, for most LDA topics (14/25), the posts associated with them contained a coherent set of parental concern categories. Figure 5 shows the parenting or family health concern categories identified for each topic. For example, the topic doctor+sick+cough+hospit contained categories all associated with children being ill or taken to the doctor: child feeling ill, COVID concerns, and fever. The topic word+speech+autism+delai likewise contained a coherent set of concerns around development: autism, developmental concerns, education questions, speech, and speech issues.

Other topics (10/25) appeared to have 2 or more distinct but closely related and co-occurring concerns, for example, the topic sleep+night+wake+feed contained the categories bottle feeding, breastfeeding, infant crying, routine, sleeping in parents’ bed, sleeping routine, nap, regression, and waking up at night. Here, sleep issues co-occur with breastfeeding, and many posts in this topic discuss breastfeeding and sleep. The topic told+behaviour+tell+punish contains posts on intimate partner violence (including child on child, adult on child, and adult on adult violence), generalized concerns around separation and children spending time with their fathers, bad behavior, and behavioral problems. While most topics contain concerns that are related or those we would expect to co-occur, the topic parti+birthdai+seat+christma is an amalgamation of concerns around celebratory events (birthdays or Christmas) and car seats (safety concerns and advice about buying).

### Assignment to Categories Identified by Lavigne et al [31]

Traditional priority-setting approaches are an important comparator for topics extracted from large web-based data sets. Here, we make detailed comparisons between our topics and those identified by Lavigne et al [31] using traditional priority-setting methods.

**Figure 6:**
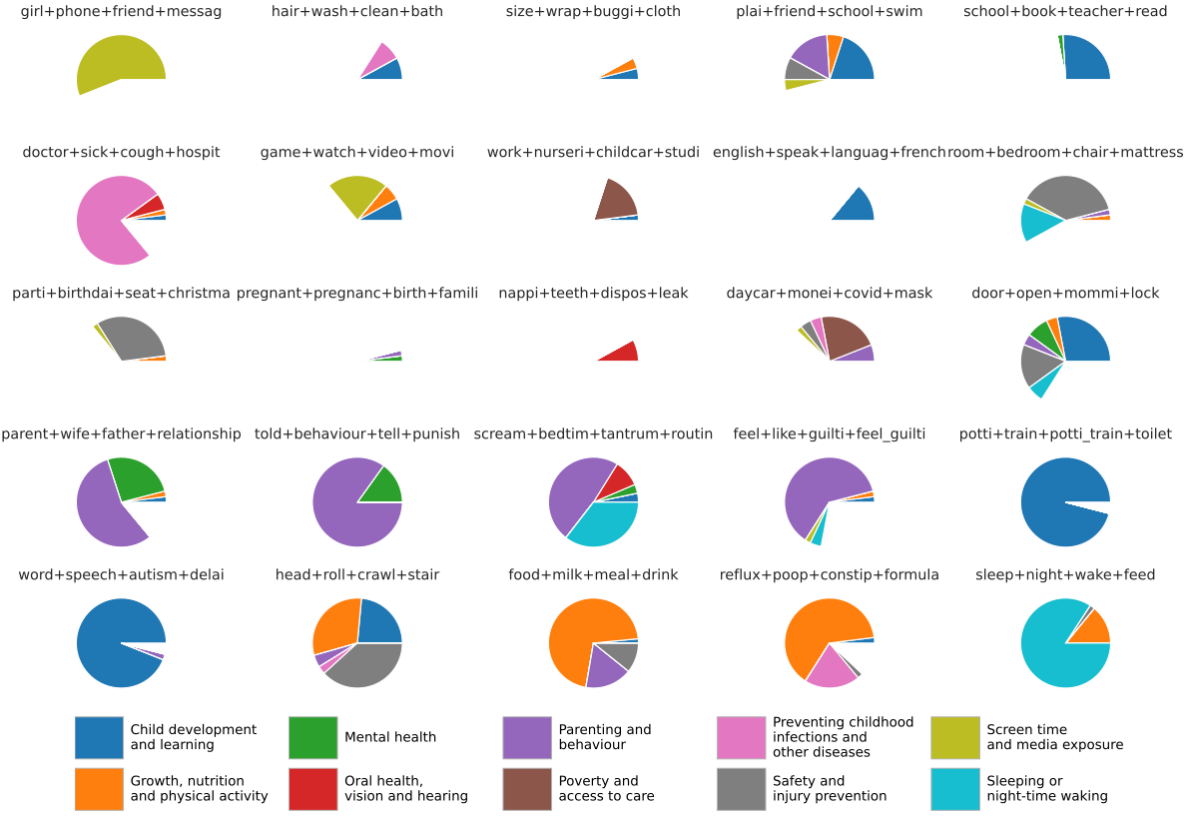
The proportion of posts in each topic assigned to the child health categories identified by Lavigne et al [31].

In 15 out of 25 of LDA topics, a majority of posts could be assigned to one or more of the parenting question categories devised by Lavigne et al [31]. Figure 6 shows the Lavigne et al [31] categories identified within LDA topics. Several topics contain a majority of posts relevant to a single category. For example, sleep+night+wake+feed contained a majority of posts within the *Sleeping or night-time waking* category. Others contained a plurality of categories, for example, the LDA topic door+open+mommi-lock is split between *Child development and learning*, *Safety and injury prevention*, *Mental health*, *Parenting and behaviour*, *Sleeping or night-time waking*, and *Growth, nutrition and physical activity*.

For 10/25 of the LDA topics, a majority of posts were not attributable to any of the categories identified by Lavigne et al [31]. In 5 out of 10 of these topics, less than 25% of posts could be attributed to a category. These topics were pregnant+pregnanc+birth+famili—a topic concerned with family planning, nappi+teeth+dispos+leak—a topic concerned with reusable nappies, size+wrap+buggi+cloth—another topic concerned with reusable nappies as well as clothing and transporting a baby, hair+wash+clean+bath—a topic concerning hygiene, and work+nurseri+childcar+studi—a topic covering balancing childcare with work and studying.

The topics identified here cover 10 out of 11 of the Lavigne et al [31] categories. *Environmental toxins* is the only one without representation. *Parenting and behaviour*, *Child development and learning*, *Growth, nutrition and physical activity*, *Sleeping or night-time waking*, *Screen time and media exposure*, and *Preventing childhood infections and other diseases* are all well represented—presenting in multiple topics and as a majority in at least one. *Mental health* and *Poverty and access to car*e are present in multiple topics without being a majority in any, and *Oral health, vision, and hearing* is present in small numbers in 3 topics.

## Discussion

### Principal Results

We have collated posts from the r/Parenting forum and the Mumsnet parenting forum and then used LDA to model the most common topics of discussion. We found that a model with 25 topics was the most coherent. An analysis of the content of posts associated with these topics indicated that most topics brought together posts on a related set of parenting concerns, and we found significant overlap between the content of our LDA topics and parenting priority categories identified using traditional priority-setting methods [31].

### Strengths and Limitations

A key strength of this work is that topic modeling using LDA allows us to use a large collection of posts, revealing the latent semantic structure present within them; however, its outputs can vary significantly depending on parameterization, and the evaluation of output quality is a contentious issue [33]. We have used one type of coherence score (C_v_) [30] to optimize our selection of the number of topics parameter. However, there are multiple scores that can be used to evaluate the coherence of the model and others that evaluate the model through its predictive ability (perplexity) [34]. While there is strong evidence that C_v_ correlates more strongly with human interpretability than other evaluation functions for certain benchmark data sets [30], there is no guarantee that our parameter selection is optimal. However, we do consider it to be functional, providing topics that we have been able to interpret through further analysis.

The additional methods used to interpret the topics identified using LDA are also a strength of this work. Applying content analysis allows the context of each post and a subjective interpretation to feed into the description of the topic, while assignment to previously established parenting priority categories [31] leverages the data and analysis of previous work. Typically, topic modeling studies use the words most associated with a topic [35], or a human interpretation of these words [36], to provide a description, but this may not always provide the reader with a complete picture of the topic. Combining LDA and qualitative analysis of a subset of posts has been used previously to provide a more detailed interpretation of topics of parent discussions of fussy eating [37] and the concerns of foster families during the COVID-19 pandemic [15].

However, our content analysis and categorization also have limitations. To reduce the labor requirements of the task, we have analyzed a sample of 50 posts from each topic (1%-16% of the total posts in each topic compared to 100 posts or 20% of posts per topic used by others [15]). Therefore, the concerns identified may not be fully representative, for example, we could be 90% confident that a concern identified 3 times in the sample of 50 would appear in 0.4%-11.5% of the posts in that topic. A topic appearing 20 times would appear in between 28.6% and 51.3% of posts. So, while the topic-specific concerns shown in Table 1 and Figure 5 are likely to occur beyond the sample, the proportions they indicate have a large margin of error, and the list is unlikely to be exhaustive. We also recognize that many posts are discarded before content analysis (those with less than 75% probability of being generated by a topic), and so the themes identified are only those present in posts with a strong affinity to the topic.

Another limitation of this work comes from the representativeness of the social media users as a sample of the wider population [38]. As there are no detailed analyses of the sociodemographics of either source available, we can only speculate on whether the views expressed on these forums represent those of the wider population (including those from significant economic deprivation).

To address this, we envision that future work will take the outputs generated here (the descriptions of the topics) and use them as material for targeted engagement with underrepresented groups such as those on low incomes. This will allow an assessment of whether the views expressed on these forums differ from those of the underrepresented groups and will allow us to bring in the voice of these groups and allow expression of their concerns. We also envision that these data sources may be used to shape future research priorities and identify unmet needs across health and childcare provision, highlighting further the importance of acknowledging the underrepresented voices.

### Comparison With Prior Work

Previous priority-setting work has identified robust parental concerns relating to childhood chronic disease [39], preventative care [31], and mental health [40]. The views collected in our analysis come from the general population, and so, the concerns found are most comparable to those previously found on preventative care and mental health.

Our results strengthen the evidence signaling parent priorities in 10 of the 11 parent priority categories presented by Lavigne et al [31] on pediatric preventative care. Lavigne et al [31] also present the top 10 most important unanswered questions from the perspective of parents and clinicians. Some of these questions are clearly reflected in the priorities identified here. For example, we identify several topics (eg, told+behaviour+tell+punish) that include behavioral problems as a priority, a priority also articulated by Lavigne et al [31] with the question “What are effective strategies for behaviour management in children?”. Concerns around the development of one’s child are strongly expressed within the word+speech+autism+delai topic echoing the question “What are effective methods for screening for developmental delay in children?” presented by Lavigne et al [31].

Both Mumsnet and Reddit have been the subject of prior research into parental concerns and pediatric or maternal health [13,18-20,37,41]. For example, previous work has highlighted the use of Mumsnet by parents of children with mental health needs [20] showing that it was used primarily to offer and receive emotional support and to find advice on methods or techniques that could be used without the help of professionals. We have also found evidence of parents seeking support for children with mental health issues (topic 23—parent+wife+father+relationship) with intergenerational mental health issues, divorce, and authoritarian parenting also found in this topic.

Previous work using Reddit as a data source has used LDA alongside thematic analysis to investigate parental discussion of fussy eating [37]. Using similar methods (but a different subreddit r/Toddler) to those presented here, this work used LDA to cluster posts into topics and then took a single topic (fussy eating) forward for further thematic qualitative analysis.

This work differs from the above by presenting an overview of discussion on 2 broadly defined parenting forums rather than a more detailed analysis of specific issues. Previous work that has taken a similarly broad approach includes an analysis of the posts on the subreddits r/Mommit and r/Daddit, again combining LDA topic modeling with qualitative analysis to give a broad overview of discussion topics and presenting a comparison between the 2 forums [23]. We build on this work by including 2 much larger data sets (99,848 posts from Mumsnet and 69,846 posts from r/Parenting) and outline methods to visualize and perform an inductive content analysis of a data set of this size. We identify many similar topics, including sleep issues, toilet training, food/fussy eating, managing multiple children/sibling conflict, talking about difficult issues, phone/social media use, and parental guilt. This indicates that there are robust concerns that emerge as topics of discussion on a range of parenting forums when this type of analysis is applied. Several additional concerns such as child hygiene, fear of child injury, and fear of being a bad parent were also identified.

Recent work has also applied LDA modeling to parenting forums such as Momsholic [42]. They found feeding, sleeping, medical problems, development, and learning to be the most discussed topics—all major topics found within our analysis. Both studies also agree that sleep is the most discussed.

### Conclusions

Here, we present further evidence on the priorities and concerns of parents through the identification of topics commonly discussed on 2 well-used parenting advice forums (Mumsnet and r/Parenting). Our results show that LDA can be used to identify common parenting concerns from large-scale parenting forum data, and alongside the content analysis outlined here can be used to provide an indicative map of concerns and priorities commonly discussed. This provides a method for the rapid identification of concerns and priorities from these, or similar, large-scale data sources. We also hope our results can be used as a signpost for others looking to use data from these sources, for example, in the development of content for targeted engagement or qualitative research with families.

## Abbreviations

LDA: latent Dirichlet allocation
